# Enrollment of Black Participants in Pivotal Clinical Trials Supporting US Food and Drug Administration Approval of Chimeric Antigen Receptor–T Cell Therapy for Hematological Malignant Neoplasms

**DOI:** 10.1001/jamanetworkopen.2022.8161

**Published:** 2022-04-20

**Authors:** Samer Al Hadidi, Carolina Schinke, Sharmilan Thanendrarajan, Maurizio Zangari, Frits van Rhee

**Affiliations:** 1 Myeloma Center, Winthrop P. Rockefeller Cancer Institute, University of Arkansas for Medical Sciences, Little Rock

## Abstract

**Importance:**

Disparities that affect Black persons with various hematological malignant neoplasms are substantial, yet little is known about disparities related to the use of US Food and Drug Administration (FDA)–approved chimeric antigen receptor–T cell (CAR-T) therapy.

**Objective:**

To examine the enrollment of Black participants in clinical trials that resulted in a subsequent FDA approval of CAR-T products in hematological malignant neoplasms.

**Design, Setting, and Participants:**

A cross-sectional study was performed using publicly available data on drug products and demographic subgroups from Drugs@fda in the period of August 2017 to May 2021. Data analysis included patients with large B cell lymphoma, follicular lymphoma, mantle cell lymphoma, acute lymphoblastic leukemia, and multiple myeloma who were enrolled into 7 clinical trials that investigated various CAR-T products. The study was conducted from July 1, 2021, to November 30, 2021.

**Main Outcomes and Measures:**

Frequencies of participation of Black participants were calculated with adjustment for disease prevalence.

**Results:**

Of the 1057 enrolled patients included in the study, CAR-T products were given to 746 patients (71%), and efficacy was reported for 729 enrolled patients (69%) across all the approved CAR-T products and indications. Most patients (1015 patients [96%]) were enrolled in the US. Black participants were included in the racial category *other* in the study that supported tisagenlecleucel approval in acute lymphoblastic leukemia; otherwise, their enrollment was specified either in the study publication and/or the demographic subgroup information available under the FDA product labeling information. The number of Black participants who received the CAR-T product and had reported efficacy varied between studies (range, 1-12 participants [2%-5%]). Adjusted prevalence measures showed the lowest participation to prevalence ratio of 0.2 for multiple myeloma and 0.6 for large B cell lymphoma.

**Conclusions and Relevance:**

The findings of this study suggest that there are substantial disparities affecting Black patients across all approved CAR-T products used to treat hematological malignant neoplasms with otherwise limited effective treatment options. The study findings might aid policy discussions regarding the immediate need of regulations that enforce certain thresholds of Black patients’ enrollment before granting FDA approval.

## Key Points

**Question:**

What is the proportion of Black participants enrolled in pivotal clinical trials supporting US Food and Drug Administration approval of chimeric antigen receptor–T cell (CAR-T) therapy for hematological malignant neoplasms?

**Findings:**

In this cross-sectional study of data from 7 clinical trials that resulted in a subsequent Food and Drug Administration CAR-T therapy approval for hematological malignant neoplasms, the proportion of Black participants, adjusted to the disease prevalence, was suboptimal.

**Meaning:**

The findings of this study suggest that disparities in the use of CAR-T therapy in clinical trials for hematological malignant neoplasms exist and are disproportionately affecting Black participants.

## Teaser

This cross-sectional study examines the enrollment of Black participants in clinical trials that resulted in a subsequent US Food and Drug Administration (FDA) approval of chimeric antigen receptor–T cell therapy products for hematological malignant neoplasms.

## Introduction

Disparities affecting Black persons are prevalent. Black individuals are underrepresented in clinical trials that support US Food and Drug Administration (FDA) drug approvals of cancer therapeutics, such as novel therapies used in different malignant neoplasms, including hematological malignant neoplasms.^1^ Chimeric antigen receptor–T cell (CAR-T) therapy is a major advancement in the treatment of hematological malignant neoplasms and is currently approved for use in multiple myeloma (MM), acute lymphoblastic leukemia (ALL), diffuse large B cell lymphoma (DLBCL), mantle cell lymphoma (MCL), and follicular lymphoma (FL).

The approval of CAR-T products in hematological malignant neoplasms is preserved for advanced, relapsed, and/or refractory disease status with otherwise no or limited available effective treatment options; thus, it is vital to ensure that enrollment of Black patients in clinical trials that study novel therapies with promising efficacy is adequate. Enrollment of Black participants in clinical trials that supported those approvals are not well described. This study assessed the frequency of Black participants in clinical trials that supported FDA-approved CAR-T products in the US.

## Methods

This cross-sectional study used publicly available data on drug products and demographic subgroups from Drugs@fda along with recent cancer statistics for Black individuals.^2^ All CAR-T therapies approved in the period of August 2017 to May 2021 were included. Prevalence-corrected estimates for enrollment of Black participants were calculated as the percentage of Black participants among clinical trial participants divided by the percentage of Black individuals in the disease population (participation to prevalence ratio [PPR]). Disease prevalence was based on disease category and the Surveillance, Epidemiology, and End Results Program data. A PPR range between 0.8 and 1.2 was considered to reflect similar representation of Black individuals in the trial and disease population.^1^ The study was conducted from July 1, 2021, to November 30, 2021. All study procedures were approved by the University of Arkansas for Medical Sciences institutional review board with a waiver of informed consent owing to the retrospective nature of data, and the study followed the Strengthening the Reporting of Observational Studies in Epidemiology (STROBE) reporting guideline for cohort studies.^3^

Different studies reported racial and ethnic groups differently. In the study^4,5,6^ that supported tisagenlecleucel approval in ALL, race was reported as Asian, White, or other, and ethnicity was reported as Hispanic or other. In the study^7,8^ that supported tisagenlecleucel approval in DLBCL, race was reported as Asian, Black or African American, White, or other with no available data on ethnicity. In the study^9,10^ that supported axicabtagene ciloleucel in DLBCL, race was reported as Asian, Black or African American, White, or other, and ethnicity was reported as Hispanic or Latino or not Hispanic or Latino. In the study^11,12^ that supported axicabtagene ciloleucel in FL, race was reported as Asian, Black or African American, or White with no available data on ethnicity. In the study^13,14^ that supported brexucabtagene autoleucel in MCL, race was reported as Black or African American, Native Hawaiian or other Pacific Islander, White, or other with no available data on ethnicity. In the study^15,16^ that supported lisocabtagene maraleucel in DLBCL, race was reported as Asian, Black or African American, White, or other, and ethnicity was reported as Hispanic or Latino or other. In the study^17,18^ that supported idecabtagene vicleucel approval in MM, race was reported as Asian, Black or African American, White, unknown, or other, whereas ethnicity was reported as Hispanic or Latino, non-Hispanic, not reported, or other.

### Statistical Analysis

The primary group in this study is African American and Black participants (hereafter, Black participants). Frequency measures were obtained from the study publication and/or the demographic subgroup information available under the FDA product labeling information.^4,5,6,7,8,9,10,11,12,13,14,15,16,17,18^ If a discrepancy in the numbers or frequencies between publication and the FDA product labeling information was noted, the FDA product labeling information was used. All statistical analyses were done using R statistical software version 4.0.5 (R Project for Statistical Computing).^19^

## Results

Approvals of 5 CAR-T products for 7 indications occurred from August 3, 2017, to March 26, 2021. Three products were approved for DLBCL (tisagenlecleucel, axicabtagene ciloleucel, and lisocabtagene maraleucel), 2 products were approved for ALL (tisagenlecleucel and axicabtagene ciloleucel), and 1 product each was approved for FL (axicabtagene ciloleucel), MCL (brexucabtagene autoleucel), and MM (idecabtagene vicleucel). All the products are approved in relapsed or refractory disease status and for patients who received multiple lines of previous therapies. Details on the labeled indication are summarized in the Table.

**Table.** Characteristics of Clinical Trials That Supported Approvals for Chimeric Antigen Receptor–T Cell Therapy in Various Hematological Malignant Neoplasms

Therapy and study	Approval date	Indication	Participants, No. (%)			Black participants to whom the product was given, No. (%)
			Enrolled set	Safety analysis set	Efficacy analysis set	
Tisagenlecleucel, Maude et al^4^	August 30, 2017	Patients up to age 25 y with B-cell precursor acute lymphoblastic leukemia that is refractory or in second or later relapse	88 (100)	68 (100)	63 (100)	NR
			Asian: 10 (11)	Asian: 6 (9)	Asian: 6 (10)	
			White: 65 (74)	White: 51 (75)	White: 46 (73)	
			Other: 13 (15)	Other: 11 (16)	Other: 11 (17)	
Tisagenlecleucel, Schuster et al^7^	May 1, 2018	Adult patients with relapsed or refractory large B-cell lymphoma after ≥2 lines of systemic therapy, DLBCL not otherwise specified, high-grade B-cell lymphoma, and DLBCL arising from follicular lymphoma	160 (100)	106 (100)	68 (100)	2 (3)
			Asian: NR	Asian: NR	Asian: 3 (4)	
			Black or African American: NR	Black or African American: NR	Black or African American: 2 (3)	
			White: NR	White: NR	White: 61 (90)	
			Other: NR	Other: NR	Other: 2 (3)	
Axicabtagene ciloleucel, Locke et al^10^	October 18, 2017	Adult patients with relapsed or refractory large B-cell lymphoma after ≥2 lines of systemic therapy, including DLBCL not otherwise specified, primary mediastinal large B-cell lymphoma, high grade B-cell lymphoma, and DLBCL arising from follicular lymphoma	111 (100)	101 (100)	101 (100)	4 (4)
			Asian: NR	Asian: 4 (4)	Asian: 4 (4)	
			Black or African American: NR	Black or African American: 4 (4)	Black or African American: 4 (4)	
			White: NR	White: 90 (89)	White: 90 (89)	
			Other: NR	Other: 3 (3)	Other: 3 (3)	
Axicabtagene ciloleucel, Palomba, et al^11^	March 5, 2021	Adult patients with relapsed or refractory follicular lymphoma after ≥2 lines of systemic therapy	123 (100)	120 (100)	81 (100)	3 (4)
			Asian: NR	Asian: NR	Asian: 2 (3)	
			Black or African American: NR	Black or African American: NR	Black or African American: 3 (4)	
			White: NR	White: NR	White: 75 (93)	
Brexucabtagene autoleucel, Wang et al^13^	July 24, 2020	Adult patients with relapsed or refractory mantle cell lymphoma	91 (100)	82 (100)	60 (100)	1 (2)
			Black or African American: NR	Black or African American: 1 (1)	Black or African American: 1 (2)	
			Native Hawaiian or other Pacific Islander: NR	Native Hawaiian or other Pacific Islander: 1 (1)	Native Hawaiian or other Pacific Islander: 1 (2)	
			White: NR	White: 75 (91)	White: 56 (93)	
			Other: NR	Other: 5 (6)	Other: 2 (3)	
Lisocabtagene maraleucel, Abramson et al^15^	February 5, 2021	Adult patients with relapsed or refractory large B-cell lymphoma after ≥2 lines of systemic therapy, including DLBCL not otherwise specified (including DLBCL arising from indolent lymphoma), high-grade B-cell lymphoma, primary mediastinal large B-cell lymphoma, and follicular lymphoma grade 3B	344 (100)	269 (100)	256 (100)	12 (5)
			Asian: 13 (4)	Asian: 11 (4)	Asian: 11 (4)	
			Black or African American: 17 (5)	Black or African American: 12 (5)	Black or African American: 12 (5)	
			White: 294 (86)	White: 232 (86)	White: 219 (86)	
			Other: 20 (6)	Other: 14 (5)	Other: 14 (5)	
Idecabtagene vicleucel, Munshi et al^17^	March 26, 2021	Adult patients with relapsed or refractory multiple myeloma after ≥4 prior lines of therapy, including an immunomodulatory agent, a proteasome inhibitor, and an anti-CD38 monoclonal antibody	140	127	100	6 (6)
			Asian: 3 (2)	Asian: 3 (2)	Asian: 2 (2)	
			Black or African American: 8 (6)	Black or African American: 6 (5)	Black or African American: 6 (6)	
			White: 113 (81)	White: 102 (80)	White: 78 (78)	
			Unknown: 10 (7)	Unknown: 10 (8)	Unknown: 9 (9)	
			Other: 6 (4)	Other: 6 (5)	Other: 5 (5)	

Abbreviations: DLBCL, diffuse large B cell lymphoma; NR, not reported.

Of the 1057 enrolled patients included in our study, CAR-T product was given to 746 patients (71%), and efficacy was reported for 729 enrolled patients (69%) across all the approved CAR-T products and indications. Most enrolled patients with reported CAR-T efficacy (1015 patients [96%]) were enrolled in the US.

In the study^4,5,6^ that supported tisagenlecleucel approval in ALL, Black participants were included in a racial category termed *others*; otherwise, their enrollment was specified mainly in the demographic subgroups information available under the FDA product labeling information. Most of the publications did not report proportions of various racial and ethnic groups. The number of Black participants who received the CAR-T product and had reported efficacy varied between studies (range, 1-12 participants [2%-5%]), with the lowest number of enrolled Black participants reported in the study for brexucabtagene autoleucel used in MCL (1 participant).^13^ The total number of Black participants enrolled with CAR-T efficacy reported per disease category was 18 (range, 2-12 participants per study) for DLBCL (total, 425 participants; range, 68-256 participants per study). For MM, 1 product was approved in the studied period, with only 6 (6%) Black participants enrolled. Frequencies of Black participants and other reported racial groups are summarized in the Table.

When adjusted to disease prevalence, for MM, which is more common among Black persons,^2,20^ adjusted prevalence measures showed the lowest PPR of 0.2, whereas for DLBCL, a better, although still suboptimal, PPR of 0.6 reflects better enrollment and lower prevalence of DLBCL among Black persons. For ALL, PPR was not calculated because of a lack of data on enrolled Black participants. The PPRs were 0.8 for FL and 0.4 for MCL (Figure).

**Figure.**
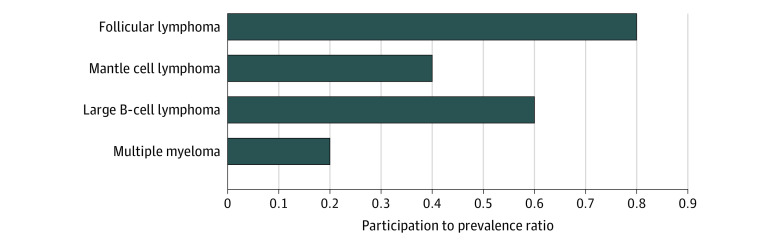
Participation to Prevalence Ratio of Black Participants Enrolled in Clinical Trials That Supported Approvals for Chimeric Antigen Receptor–T Cell Therapy in Various Hematological Malignant Neoplasms

## Discussion

This cross-sectional study found that Black persons were underrepresented in clinical trials that supported CAR-T therapies for various hematological malignant neoplasms. This disparity was evident across all studied products and across all indications, although it was most evident for MM.

The incidence of MM is more than 2 times higher among Black persons than among non-Hispanic White persons.^20,21^ Black patients with MM face multiple disparities, including lower use of hematopoietic stem cell transplantation, palliative care, and novel therapeutics, which may result in worse outcomes.^22,23^ Lack of access to novel therapies that disproportionately affect Black persons may result in further widening of the existing established disparities.^24,25^ Implementing the least acceptable race-specific enrollment percentage maybe a potential solution for registration trials.^24^ A 10-year implementation plan is feasible and can be entertained by the FDA to help improve enrollment of Black persons across all cancers and, specifically, for MM and other malignant neoplasms that are more common among Black persons.^24^

Despite the low numbers of Black participants in trials for FL, the PPR was 0.8. This is likely because FL is less common than other hematological cancers among Black persons. Matching enrollment to disease prevalence will be the appropriate way to ensure ideal enrollment across various racial groups and will provide a reflection of actual population data.

Access to clinical trials and, specifically, CAR-T clinical trials maybe limited. Collaboration among national societies, accreditation organizations, and regulatory agencies may help in improving access to CAR-T products.^26^ Out-of-pocket costs for patients and caregivers, as well as distance to the treatment center, may disproportionately affect patients with lower socioeconomic status and should be addressed.

Multiple ongoing and recently published studies^10,27^ suggest a possible benefit of CAR-T products when used at an earlier treatment stage in patients with DLBCL. Such use in earlier disease settings should draw more attention to the disparities reported by our study to avoid further worsening.

Most of the demographic data were reported in the FDA package insert and rarely were available in the publications in medical journals. We believe that such a lack of transparency in reporting demographic data in medical journal publications of our analyzed CAR-T studies is problematic and should be discouraged.

### Limitations

This study has some limitations. Because this was a cross-sectional study, it cannot establish specific factors that led to low enrollment of Black persons. Most of the approved products were based on low number of clinical trials and enrolled participants. We were not able to investigate access to centers with open clinical trials, which may limit enrollment of Black persons and exacerbate established disparities. Nevertheless, to our knowledge, our study is the first to report on disparities in the field of CAR-T products that are used in difficult to treat hematological malignant neoplasms.

## Conclusions

CAR-T therapy represents an important recent advancement in the oncology field. The findings of this study suggest that low enrollment of Black persons exists in trials for CAR-T therapy and that the disparity is substantial and ongoing, especially for therapies to treat MM. Efforts should be made to understand and overcome barriers that lead to decreased enrollment of Black participants in clinical trials that include novel, potentially beneficial, and/or curative CAR-T therapies in difficult-to-treat hematological malignant neoplasms with otherwise limited treatment options. This may include allowing more medical centers that serve a higher percentage of Black participants to be part of the currently ongoing CAR-T therapy clinical trials.
